# Dispensing of antibiotics for tuberculosis patients using standardized patient approach at community pharmacies: results from a cross-sectional study in Pakistan

**DOI:** 10.3389/fpubh.2023.1241551

**Published:** 2024-01-08

**Authors:** Ali Hassan Gillani, Hafsa Arshad, Hasan Mujtaba, Muhammad Farooq Umer, Sen Xu, Wenjing Ji, Kamran Bashir, Jie Chang, Caijun Yang, Yu Fang

**Affiliations:** ^1^Department of Pharmacy Administration and Clinical Pharmacy, School of Pharmacy, Xi’an Jiaotong University, Xi’an, Shaanxi, China; ^2^Center for Drug Safety and Policy Research, Xian Jiaotong University, Xi’an, Shaanxi, China; ^3^Shaanxi Centre for Health Reform and Development Research, Xi’an, Shaanxi, China; ^4^Department of Pathology, Shaheed Zulfiqar Ali Bhutto Medical University, Islamabad, Pakistan; ^5^College of Dentistry, King Faisal University, Hofuf, Alahsa, Saudi Arabia; ^6^College of Pharmacy, University of Sargodha Pakistan, Sargodha, Pakistan

**Keywords:** dispensing of antibiotics, tuberculosis patients, dispensing practices, simulated client approach, community pharmacies

## Abstract

**Background:**

Pakistan is among top countries for antibiotic consumption around the globe and patients often receive antibiotics directly from community pharmacies. Thus, our aim was to assess the drug dispensing practices of pharmacies for presumed and confirmed tuberculosis by using standardized patients’ method in Pakistan.

**Methods:**

In this cross-sectional study, we adopted two standardized patient cases in pharmacies of three cities of Punjab. The first case involved a presumed tuberculosis patient presenting with 2–3 weeks of cough and fever (Case-1), and the second case involved a confirmed tuberculosis patient carrying microbiologically confirmed tuberculosis results (Case-2). The ideal management for Cases-1 and Case-2 is referral of standardized patients to a healthcare provider without dispensing antibiotics or steroids, or both. The differences in antibiotic use, steroid use, and the number of medicines dispensed in referred and non-referred patients between Case-1 and Case-2 were analyzed using descriptive statistics.

**Results:**

Between April 1, 2020, and July 31, 2020, standardized patients completed 575 out of 598 interactions among community pharmacies in Lahore, Rawalpindi, and Sialkot. We recorded ideal management in 115 (37.7%) of the 305 Case-1 interactions and 130 (48.1%) of the 270 Case-2 interactions. Antibiotic dispensing was higher in Case-1, with 71 out of 305 instances (23.3%), than in Case-2 interactions, with 27 out of 270 instances (10.0%). Anti-tuberculosis drugs were dispensed to 1 patient in Case-1 (0.3%) and to 19 patients (7.0%) in Case-2.

**Conclusion:**

Slightly more than one-third of pharmacies in Punjab, Pakistan, ideally managed patients with presumed tuberculosis, but almost half of them ideally managed cases of confirmed tuberculosis. The presence of confirmed diagnosis slightly changes the behavior in the correct management of patients.

## Introduction

Tuberculosis (TB) the second most lethal infectious disease killer after COVID-19 around the world in 2021, with approximately 1.6 million people died from TB in 2021 (1). Among the 22 countries in the Eastern Mediterranean region, Pakistan ranks 6th in terms of TB disease burden and 1.5 million among 210 million suffers from TB (2). Antibiotic resistance (AR) is increasing drastically around the globe and Pakistan is no exception with the increase of 65% in antibiotic consumption between the years 2000 and 2015, with the number of DDDs rising from 0.8 to 1.3 billion (3, 4). In TB patients its emergence is elevating due to various factors, including delays in diagnosis, inappropriate drug regimens, treatment costs, treatment discontinuation and incomplete courses, poor follow-up, and lack of social support (5). A time series analysis conducted from 2000 to 2017 in the Eastern European region and Central Asian region revealed the emergence of multidrug-resistant tuberculosis (MDR-TB), particularly AR, in treatment regimens (6). In lower-income countries, antibiotics used in the treatment of TB are readily accessed through community pharmacies, and community pharmacies are serving as the primary point of contact for initial TB care (7). TB patients often seek medical care from private health sectors, private practitioners, clinics, NGOs, and community pharmacies. At the initial stage, TB symptoms such as cough, chest pain, and fever may lead many TB patients to approach community pharmacies for temporary relief, which can ultimately contribute to the emergence of AR (8).

The involvement of pharmacists at community pharmacies in TB management is crucial in combating AR in TB. The dispensing of antibiotics at community pharmacies raises concerns in TB management, as antibiotics are often dispensed without a prescription and in the absence of a qualified pharmacist (9). Study from South Africa, India, Uganda, Vietnam, Zambia, and Pakistan revealed that approximately 60% of TB patients seek assistance from community pharmacies before receiving a proper diagnosis of TB (10–12). The dispensing of broad-spectrum antibiotics to TB patients is a major contributing factor to the development of MDR-TB (13). While the drug regulatory authority of Pakistan has mandated the presence of a qualified pharmacist at community pharmacies for antibiotic dispensing in TB cases, the common practice of dispensing antibiotics without prescription still persists in Pakistan (14, 15).

The dispensing practices of antibiotics can be monitored through the use of the standardized client method. Also known as the simulated or mystery client method, this approach is considered the gold standard tool for monitoring the dispensing practices of antibiotics worldwide (16). A simulated study conducted in South Africa, India, China, and Vietnam yielded promising results in identifying the dispensing practices of antibiotics in TB (17–20). The mystery client method is highly effective approach for observing the dispensing practices of antibiotics in TB, as many patients initially seek care at community pharmacies (21). The use of standardized patient (SP) surveys is an ideal method to assess the clinical and dispensing skills in real-life settings at community pharmacies. Mystery shoppers, acting as trained actors following predefined standardized protocols, evaluate the actual dispensing practices and counseling skills of qualified personnel in various disease management scenarios (10). In Pakistan, community pharmacies are extensively distributed and offer healthcare services, with 96% of them dispensing antibiotics as over-the-counter (OTC) drugs without a prescription. As a result, community pharmacies play a crucial role in TB management but also contribute to the development of MDR-TB due to inappropriate dispensing of antibiotics (22). As part of the “WHO End TB Strategy” program, the World Health Organization (WHO) has implemented the National TB control program in Pakistan, giving top priority to community pharmacies and private sector stakeholders to promote TB control. However, despite the concurrent efforts of the government and the availability of national TB guidelines, there is still a misuse of antibiotics in TB management in Pakistan (23). The most common dispensing practice in TB at community pharmacies is the provision of fluoroquinolones and corticosteroids for temporary relief of symptoms (24). Previous cross-sectional and qualitative studies have reported the irrational dispensing of antibiotics in TB in lower-income countries (25, 26). A study conducted in China reported the non-prescription dispensing of antibiotics for upper respiratory tract infections and diarrhea by SP (21). Simulated patient surveys have been conducted in both lower-income and high-income countries to monitor the dispensing practices of antibiotics at community pharmacies. These surveys are widely accepted, cost-effective, and can be completed within a limited time frame (11, 27). To the best of our knowledge, there is a lack of studies utilizing the SP approach to evaluate the dispensing practices of antibiotics in TB management at the community level in Pakistan. The aim of our study is to assess the non-prescription dispensing of antibiotics through simulated patients at community pharmacies.

## Materials and methods

### Study design and setting

This cross-sectional study was conducted in three cities of Punjab, Pakistan: Lahore, Rawalpindi, and Sialkot. We targeted community pharmacies in these cities due to their high number and availability of pharmacists. Where there were multiple pharmacists employed at pharmacy, we attempted to include all of them in the study at different time frames. If that wasn’t possible, we randomly selected one pharmacist. The study evaluated the drug dispensing practices and medical advice provided by pharmacies toSPs presenting either Case 1 (presumptive TB) or Case 2 (confirmed TB). We aimed to assess the extent of antibiotic misuse resulting from the lack of disease confirmation (Case 1) versus the inappropriate use of antibiotics in confirmed cases (Case 2). We established a benchmark based on national guidelines for TB control in Pakistan and the International Standards for Tuberculosis Care. According to these guidelines, pharmacists should counsel TB patients and refer individuals with TB symptoms to the nearest public or private health facilities for testing (28, 29). Pharmacists adhering to these guidelines should refer the SPs to healthcare providers (HCPs) rather than dispensing antibiotics or steroids, which are prescription drugs. Given the similarity of TB symptoms to those of COVID-19, and considering the study was conducted during the time and region affected by COVID-19, the SPs carried COVID-19 negative test results and presented them to the pharmacist upon request to rule out the possibility of a COVID-19 diagnosis.

SPs visited a total of 189 pharmacies in Lahore, 101 pharmacies in Rawalpindi, and 41 pharmacies in Sialkot. Convenience sampling was employed to select these pharmacies from 28 different low and middle-income localities in Lahore, 17 localities in Rawalpindi, and 12 localities in Sialkot. Data collection took place from April to July 2020. In Lahore, a total of 330 interactions were completed (180 for Case 1 and 150 for Case 2), while in Rawalpindi, 175 interactions were conducted (90 for Case 1 and 85 for Case 2). In Sialkot, 70 interactions were performed (35 for Case 1 and 35 for Case 2). We tried to target same pharmacies for case 1 and 2.

### Standardized patients

SPs were recruited from general public and common layman people were selected as data collectors. This is done to make the situation more realistic. The SPs were recruited through controlled advertisement and principal investigator himself did the screening for the eligibility of SPs. A total of 16 SPs were made it to the training section and after 2 weeks of training and dry runs we shortlisted the 11 persons (6 to present Case 1 and 5 to present Case 2). Among the 11 SPs 7 were males and 4 were females, 6 were undergraduate and 5 were postgraduate, 9 had family income less than 50,000 PKR and 2 had income exceeding 50,000 PKR.

The SPs trained to present Case 1 visited pharmacies with symptoms of cough and fever persisting for 2–3 weeks, directly seeking drugs from the pharmacy. The possible differential diagnoses for Case 1 included pneumonia, upper respiratory tract infection, acute or chronic bronchitis, and asthma. While some of these conditions may warrant antibiotic use, it should be noted that antibiotics should not be dispensed without a physician’s prescription. On the other hand, the SPs trained to present Case 2 exhibited symptoms of 1 month of cough and fever with a positive TB sputum smear test from a government dispensary. In Case 2, TB was confirmed, although the SPs stated that they did not fully understand the contents of the test report. This situation allowed the pharmacist to make the correct diagnosis and realize that short-term antibiotics would not be helpful. However, they still offered antibiotics because they were unaware of the test results. After each pharmacy visit, the SPs were debriefed using an exit questionnaire within 1 h of the visit.

The data in the observations and during interactions was first transferred to the exit questionnaire and it was uploaded in Excel and SPSS file to make the data analyzable.

### Exit questionnaire

Exit questionnaire consisted of following

- The cover page includes the following information: form number, facility ID, pharmacist ID, pharmacy details, visit details, date of interaction, start and finish time of interaction, as well as other data pertaining to the features of the visit.
- The pharmacist inquired about history-related questions, which were particular to the case and presented in a numbered format, with the inclusion of a “other” choice.
- Any clinical or physical examinations attempted (generally not case-specific and enumerated with “other” option).
- Diagnostic tests ordered (case-specific and enumerated with “other” option).
- Whether diagnosis (and details if mentioned), referral (and details if specific), and “return to provider” instructions (and details) were provided.
- Medicines prescribed and dispensed, including price, quantity, place of purchase, and Anatomical therapeutic classification (ATC) code when possible.
- Prices charged for consultation, labs, and medicines, itemized when possible and aggregated with notes when not.

The questionnaire is added as Supplementary material.

### Standardized patient case descriptions

#### Case 1

##### Case description

First case of presumed TB symbolized 2–3 weeks of cough with low grade fever and directly seeking care from pharmacist.

##### Presentation of standardized patient

The case was presented with the statement, “Dear, I am suffering from cough and fever that is not getting better. I have 2–3 weeks cough with high frequency at night and early morning, accompanied by on-and-off, low-grade fever. I am producing sputum but without any blood. I also lost of appetite and loosening of clothes.” If the pharmacist asked about taking medicines for this illness, the patient would say no.

##### Expected case management

Written or verbal referral to a DOTS facility or an HCP without dispensing any antibiotics.

#### Case 2

##### Case description

Chronic cough that is accompanied by positive sputum smear report for TB from a government hospital and directly seeking care from pharmacist.

##### Presentation of standardized patient

Case 2 was presented with the opening statement, “Sir, I am suffering from cough for almost a month and also have fever.” The patient further continues while showing positive sputum results to the pharmacist “I went to the government hospital laboratory and they get my sputum tested. I have these test results. I lost appetite and loosening of clothes if prompted by the pharmacist. Can you please give me some medications?” At that time, this case has had a cough for 1 month and produces sputum without blood, with low grade fever for the same duration as cough. If the pharmacist asked about taking medicines for this illness, the patient would say no.

##### Expected case management

Written or verbal referral to a DOTS facility or a doctor without dispensing any antibiotics (including anti-TB drugs and fluoroquinolones) or steroids.

### Statistical analysis

Our unit of analysis was whether the patient was ideally managed from a TB perspective, consistent with Standards guidelines for TB Care in Pakistan and International Standards for TB Care. We regarded written or verbal referral to HCP as ideal management for both cases, without dispensing any antibiotics, including anti-TB drugs and steroids or fluoroquinolones. We calculated the proportion for our primary outcome, interactions that resulted in ideal case management, interactions resulting in antibiotic, fluoroquinolone, and steroid dispensing. All analyses were done using SPSS (version 21).

### Ethical approval

We obtained ethical approvals from the medical research ethics committee of Xi’an Jiaotong University China (XJTMD11-2020), and research ethical review board committee of The Superior University, Lahore (ERB-PH2020). A waiver was granted by institutions from obtaining informed consent from pharmacies in three cities of Punjab in order to minimize Hawthorne effect. All individuals who acted as SPs were trained to protect themselves from any harmful medical interventions, such as avoiding invasive tests, injections or consuming any drugs at the pharmacy.

## Results

One hundred seventy-one (56.0%) of pharmacists referred Case 1 to HCPs, but because in 58 (33.9%) of 171 cases the SP was also given any medication, so ideal case management occurred in 115 (37.7%) of 305 Case 1 interactions. Antibiotics were dispensed in 71 of 305 instances (23.3%), steroids in 46 (15.1%), and fluoroquinolones in 18 (5.9%). OTC drugs (e.g., ibuprofen or cetirizine) use was amazingly very low 42 (13.8%) of total case 1 occasions. The dispensing of Schedule G drugs was nil (0 [0%] of 305) but there was only one case for TB drugs (1 [0.3%]).

In contrast to Case 1, 215 (79.6%) of 270 pharmacies referred Case 2 to HCPs. As before, some SPs received medications even with a referral, so ideal case management was observed in 130 (48.1%) of 270 interactions. Antibiotics was used in 27 (10.0%) of 270 occasions, steroids in 19 (7.0%), and fluoroquinolones in 29 (10.7%), OTC drugs were still dispensed in 28 (10.4%) of 270 interactions. But amazingly anti-TB drugs were given in 19 (7.0%) of total instances. Table 1 provides the percentage of the key outcome variables in three cities combined for Case 1 and Case 2.

**Table 1:** Management of Case 1 and Case 2 of all cities of Lahore, Rawalpindi and Sialkot giving frequency/proportion (%).

No.		Lahore		Rawalpindi		Sialkot	
		Case 1	Case 2	Case 1	Case 2	Case 1	Case 2
1	Number of interactions	180	150	90	85	35	35
2	Referral	134 (74.4)	150 (100.0)	36 (40.0)	50 (58.8)	1 (2.8)	15 (42.8)
3	Ideal case management	89 (49.4)	100 (66.6)	26 (28.9)	24 (28.2)	0 (0)	6 (17.1)
	Drugs						
4	Antibiotics	24 (13.3)	2 (1.3)	28 (31.1)	25 (29.4)	19 (54.2)	0 (0)
5	Steroids	21 (11.7)	9 (6.0)	17 (18.8)	7 (8.2)	8 (22.8)	3 (8.6)
6	Antibiotics plus steroids	3 (1.7)	12 (8.0)	5 (5.5)	4 (4.7)	4 (11.4)	4 (11.4)
7	Fluoroquinolones	6 (3.3)	20 (13.3)	8 (8.9)	5 (5.9)	4 (11.4)	4 (11.4)
8	OTC	37 (20.5)	0 (0)	5 (5.5)	22 (25.9)	0 (0)	6 (17.1)
10	Tuberculosis	0 (0)	7 (4.7)	1 (1.1)	0 (0)	0 (0)	12 (34.2)
11	Schedule G	0 (0)	0 (0)	0 (0)	0 (0)	0 (0)	0 (0)

**Figure 1:**
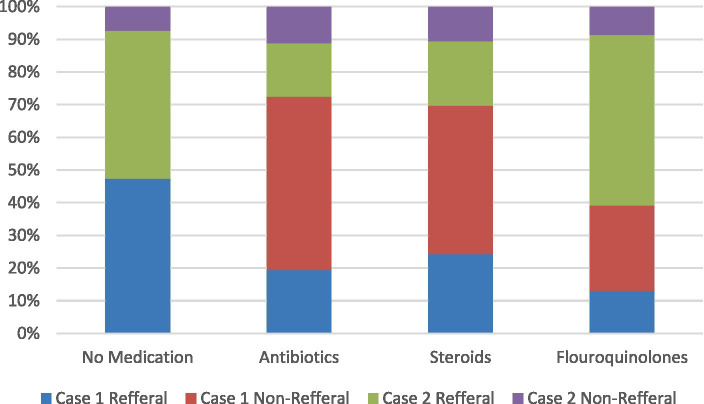
Drug use by referral decisions for two standardized patient cases. Each panel demonstrates the drug use in each case. The first panel shows cases where no drugs were given, the second panel shows cases where antibiotics were dispensed, the third panel shows cases where steroids were dispensed, and the fourth panel shows cases where fluoroquinolones were dispensed. All referral and non-referral cases for both Case 1 and Case 2 are presented in percentages. Percentages indicate the number of all cases with referrals and non-referrals, with the use of no drugs, antibiotics, steroids, and fluoroquinolones. Percentages may add up to more than 100% due to rounding.

**Figure 2:**
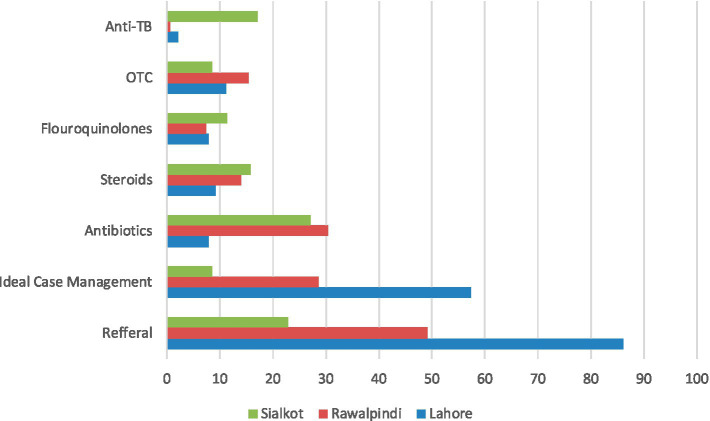
Management Case 1 and Case 2 combined by city. Referral is case in which the pharmacy staff recommended the standardized patient to seek further care from an HCP. Ideal case management in both cases is referral of SP without the dispensing of any medication.

In terms of referral, significant differences existed between Case 1 and Case 2, with a larger proportion of Case 2 being referred to HCPs. Figure 1 shows the proportions of interactions that received antibiotics, steroids, fluoroquinolones, or no drugs, separated by case and referral decision. We observed variable response patterns across the three cities, with high rates of referrals and ideal case management observed in Lahore. There were two notable differences: the use of antibiotics and steroids was high in Islamabad, while the use of fluoroquinolones and anti-TB drugs was much higher in Sialkot (Figure 2).

In terms of the number of drugs given, for Case 1, pharmacies dispensed an average of 1.62 medicines. The most commonly dispensed drugs were antibiotics [71/305 (23.3%)], followed by steroids (15.1%) and OTC medications (13.8%). Among antibiotics, the most commonly dispensed were amoxicillin (36/305, 11.8%) and Cefixime (24/305, 7.9%). Eighteen pharmacies (5.9%) dispensed fluoroquinolones (such as levofloxacin, ciprofloxacin, ofloxacin), while 46 (15.1%) of 305 gave steroids such as prednisolone and betamethasone.

For Case 2, pharmacies dispensed an average of 1.42 drugs. The drugs dispensed for this case were similar to Case 1 but with lower frequencies, except for fluoroquinolones and Anti-TB drugs, which increased to 10.4% from 5.9 and 7.0% from 0.3%, respectively.

## Discussion

To the best of our knowledge, this study is the first of its kind to employ the SP method to evaluate the treatment patterns of TB patients by pharmacies in Punjab, Pakistan. The use of SPs allowed for consistent presentation of the underlying condition across different HCPs (20, 21). The study results are valid, reliable, and comparable across pharmacies. Our key findings indicate that one patient presenting Case 1 and 19 SPs with confirmed diagnoses were given first-line anti-TB drugs. This raises concerns about the dispensing of anti-TB medications by pharmacies in major cities (Lahore and Sialkot), as pharmacies may be a potential source of irrational dispensing practices contributing to MDR-TB. In a study in Pakistan 96.9% pharmacies and medical stores dispensed antibiotics without asking for prescription and only 3.1% of pharmacies not agreeing to dispense antibiotics. The most frequently dispensed antibiotic was ciprofloxacin (22.1%) (30) Further research is needed to understand the reasons behind the dispensing of TB drugs by pharmacies, considering that their dispensing is strictly supposed to be on a prescription basis. These findings contrast with a study conducted in India where no cases were dispensed anti-TB drugs (18). Despite India and Pakistan having similar demographic distributions, there is a notable difference in regulations. In India, since 2013, regulations have been strengthened, specifically for anti-TB drugs and certain fluoroquinolones (such as moxifloxacin and levofloxacin), which are listed on Schedule H1. These drugs require a prescription from a qualified HCPs, and there is a mandatory register to record patient information, prescriber details, and the quantity of drugs supplied (18).

Although 23.3% of patients presenting with TB symptoms but without test results were dispensed medications, the use of fluoroquinolones (5.9%) and steroids in 15.1% of interactions is particularly concerning. These drugs can potentially delay the diagnosis of TB, which is alarming (31). Fluoroquinolones are essential for MDR-TB treatment, making their misuse a grave concern (31). The wide use of antibiotics and steroids for respiratory ailments has led to an increase in community-acquired infections. Unnecessary use of fluoroquinolones contributes to diarrheal illness and the development of resistant Gram-negative enteric bacteria, particularly in Pakistan and India (32). Our study also observed high utilization of amoxicillin (aminopenicillins) and azithromycin (macrolides) for respiratory ailments, which can contribute to the development of resistant strains among common respiratory pathogens like *Haemophilus influenzae and Streptococcus pneumoniae* (33). The use of steroids can increase the risk of cellulitis, herpes zoster, candidiasis, and other respiratory tract infections, as well as potentially delaying the diagnosis of TB. Furthermore, we observed a significantly higher utilization of anti-TB drugs and steroids in Sialkot compared to Lahore, indicating differences across cities. Despite our efforts to capture a diverse sample across these three cities, the variation remains unexplained. Similar findings were observed in a previous study conducted in Patna, India, where there was an increased use of antibiotics and steroids (18).

The information provided by patients to pharmacists plays a crucial role in the management of TB and AR. In the case of Case 2, which involved confirmed diagnoses, there was a noticeable increase in ideal case management, indicating that pharmacists may face challenges in diagnosing the condition. However, the change in management in confirmed cases suggested that pharmacists tend to decrease the use of medicines once a diagnosis is confirmed. The reasons behind why some pharmacies dispensed antibiotics while others did not, and why pharmacy personnel are reluctant to follow drug use regulations in these three cities, remain unknown. Better training on TB symptoms and promoting early referrals for patients with TB symptoms could also be helpful in addressing this issue. Additionally, a qualitative evaluation of pharmacists is necessary, as past studies have suggested that various factors may play a role, including local providers’ business models, pharmaceutical industry marketing practices, and patient demand for medicines (34). WHO in its end of TB work recommended TB preventive treatment for people living with HIV, household contacts of those confirmed TB, and risk groups. Globally in 2021, TB preventive treatment was provided to 3.5 million people, slightly higher from 3.2 million in 2020 (1). In order to tackle the emergence of AR and irrationality of antibiotic use AMS is one way forward. AMS refers to acceptance and implementation of interventions to reduce antimicrobial use and bacterial resistance. This program promote surveillance to follow prescribing guidelines and to promote CME among HCPs. The implementation of AMS is associated with reduced antibiotic use, fewer new prescriptions and higher physician satisfaction. Moreover, a US study showed a significant reduction in the number of unnecessary antimicrobial prescriptions after adopting AMS programs in their emergency department (35).

Our study has strengths such as firstly, demonstrated the effectiveness of the SP method in identifying inappropriate antibiotic use for TB patients. Secondly, SP method is gold standard procedure for the evaluation of practices related to any behavior in healthcare system. By using SP method, we concluded that the dispensing behavior varies as we move from unconfirmed to confirmed disease. By developing 2 different SP case presentations, we studied how providers dealt with various stages of TB disease and varying levels of diagnostic certainty our large-scale, 3-city quality of TB care at pharmacies provides accurate estimates of provider behavior that may inform not only quality-improvement efforts in health but also interventions to improve TB care and reduce transmission in the community. It also has some limitations such as it does not provide insights into the actions taken by pharmacists when they encounter regular clients or clients who return after ineffective treatment. Second, our cases are designed as one-time interactions, and the SP data do not reflect follow-up visit pathways, which have been shown by other studies. This study was conducted during the COVID-19 pandemic and findings should be considered within this unique context.

## Conclusion

High antibiotic use was observed in both cases, and in the case of Case 1, this could be potentially delaying the diagnosis. Pharmacies also dispensed anti-TB drugs, stronger fluoroquinolone antibiotics, and steroids, although in low frequencies. However, no drugs from restricted classes were given. Interestingly, there was a sharp decrease in antibiotic use in the case of confirmed diagnosis. These findings highlight the need for interventions to actively involve pharmacies in TB control and promote antimicrobial stewardship practices.

## Data availability statement

The original contributions presented in the study are included in the article/Supplementary material, further inquiries can be directed to the corresponding authors.

## Ethics statement

We obtained ethical approvals from the medical research ethics committee of Xi’an Jiaotong University China (XJTMD11-2020), and research ethical review board committee of the Superior University, Lahore (ERB-PH2020). The studies were conducted in accordance with the local legislation and institutional requirements. The ethics committee/institutional review board waived the requirement of written informed consent for participation from the participants or the participants’ legal guardians/next of kin because it was observational study of standardized patients so getting approval would tip of the Pharmacist and increase detection rate of SP.

## Author contributions

AG, HA, and YF conceptualized the study. AG, HA, HM, MU, SX, WJ, KB, JC, and CY, conducted simulated client training and collected the data. AG and HA analyzed the data. AG and HA wrote the initial draft. YF supervised the whole study. All authors contributed to the article and approved the submitted version.
