# Supplementary material for: Dispensing of antibiotics for tuberculosis patients using standardized patient approach at community pharmacies: results from a cross-sectional study in Pakistan
Source: Front Public Health. 2024 Jan 8;11:1241551. doi: 10.3389/fpubh.2023.1241551 (PMC10801376; doi:10.3389/fpubh.2023.1241551)
Supplement: Supplementary file 1 [file Table_1.DOCX]

**Exit Questionnaire**

1. **Pharmacist ID: _____________ 2. Form No: ________**
2. **City Name: ______________ 4: City ID: _________**

**5. Pharmacist name and address: ______________________________**

**6: Date of survey of each Visit: Visit 1 Visit 2 Visit 3**

**Date: ___________ ___________ ___________**

**7. SP name: ___________ ___________ ___________**

**8. SP ID ___________ ___________ ___________**

**9. Completion of the case (Yes) ___________ ____________ ____________**

**10. If no then why: ____________ ____________ ____________**

| **No** | **Questions** | **Asked Yes =1**  **N0=1** | **If not Given by SP.**  **Yes =1**  **N0=1** |
| --- | --- | --- | --- |
| 1 | Did the pharmacist advice SP to consult a provider? |  |  |
| 2 | Where did he advise the SP to go to?  Private provider=1, Government hospital =2 |  |  |
| 3 | Did the SP insist second time to get medicines? |  |  |
| 4 | Did the pharmacist advise again to consult a provider? |  |  |
| 5 | Where did he advise the SP to go to?  Private provider=1, Government hospital =2 |  |  |
| 6 | Did the pharmacist give medicine? |  |  |
| 7 | Did the pharmacist ask about duration of cough? |  |  |
| 8 | Did the pharmacist ask whether sputum is produced? |  |  |
| 9 | Did the pharmacist ask if you had TB in the past? |  |  |
| 10 | Did the pharmacist ask if anyone in the family have had  TB? |  |  |
| 11 | Did the pharmacist ask about Blood in the sputum? |  |  |
| 12 | Did the pharmacist ask about Fever? |  |  |
| 13 | Did the pharmacist ask about type of fever (low grade  vs high grade) |  |  |
| 14 | Did the pharmacist ask about chest pain? |  |  |
| 15 | Did the pharmacist ask about any loss of appetite? |  |  |
| 16 | Did the pharmacist asked have you lost weight? |  |  |
| 17 | Did the pharmacist ask about any wheezing? |  |  |
| 18 | Did the pharmacist ask about any difficulty in breathing? |  |  |
| 19 | Did the pharmacist ask anything about smoking? |  |  |
| 20 | Did the pharmacist ask anything about alcohol? |  |  |
| 21 | Did the pharmacist ask have you taken any medication for your illness? |  |  |
| 22 | Did the pharmacist ask anything about diabetes? |  |  |
| 23 | Did the pharmacist ask anything about HIV-AIDS? |  |  |
| 24 | Did the pharmacist ask your age? |  |  |

| **No** | **Physical Examination by the Pharmacist** | **Yes=1**  **No=2** |
| --- | --- | --- |
| 1 | Pulse rate |  |
| 2 | Auscultation |  |
| 3 | Temperature |  |
| 4 | Throat examination |  |
|  | **Recommended Investigation ordered by the Pharmacist** |  |
| 1 | Chest XRAy |  |
| 2 | CT Scan |  |
| 3 | Sputum Smear test |  |
| 4 | Sputum Gene Xpert Test |  |
| 5 | Sputum culture test and Drug susceptibility test |  |
| 6 | Blood total count, Differential count, ESR |  |
| 7 | Blood Diabetes Test |  |
| 8 | Blood TB-Gold |  |
| 9 | Blood TB Elisa |  |
| 10 | Mantoux test |  |
|  | **Diagnosis** |  |
| 1 | Did the pharmacist mention about the suspicion of TB in the whole conversation? |  |
| 2 | Did the pharmacist give diagnosis? |  |
|  | If yes what was the diagnose? |  |

| **Treatment** | | | | | |  |  |  |
| --- | --- | --- | --- | --- | --- | --- | --- | --- |
| **No** | **Total time taken by the Pharmacist** | | | | | **H: M: S:** | | |
|  | **Did the provider dispensed any medicine?** | | | | | | | |
|  | **Medicine Dispensed** | **Type of med**  **Tab=1**  **Cap=2**  **Syrup=3**  **Injection=4**  **Powder=5** | **Dose** | **Frequency** | **Duration** | **How many days in week** | **How many weeks** | **Drug classification code** |
| 1 | Generic |  |  |  |  |  |  |  |
|  | Brand |  |  |  |  |  |  |  |
| 2 | Generic |  |  |  |  |  |  |  |
|  | Brand |  |  |  |  |  |  |  |
| 3 | Generic |  |  |  |  |  |  |  |
|  | Brand |  |  |  |  |  |  |  |
| 4 | Generic |  |  |  |  |  |  |  |
|  | Brand |  |  |  |  |  |  |  |
| 5 | Generic |  |  |  |  |  |  |  |
|  | Brand |  |  |  |  |  |  |  |
| 6 | Generic |  |  |  |  |  |  |  |
|  | Brand |  |  |  |  |  |  |  |
| 7 | Generic |  |  |  |  |  |  |  |
|  | Brand |  |  |  |  |  |  |  |

| **No** |  | **Yes=1**  **No=2** |
| --- | --- | --- |
| 1 | How much money you paid at the end of the consultation? _____ |  |
| 2 | Did pharmacist give a receipt? |  |
| 3 | Did pharmacist offer an injection? |  |
| 4 | Did the pharmacist inform about the any side effects of the disease? |  |
| 5 | Did pharmacist speak about the cough hygiene? |  |
| 6 | Did the pharmacist speak about smoking cessation? |  |
| 7 | **Did the pharmacist ask patient to comeback?** |  |
| A | If the symptom persists |  |
| B | If symptoms become worse |  |
| C | To get medicine |  |
| D | To show the test results |  |
| E | Other |  |
| 15 | Any other questions not given in the previous list |  |
| A |  |  |
